# Framework to prioritize health outcomes of particulate matter exposure using national claims data

**DOI:** 10.1371/journal.pone.0336511

**Published:** 2025-12-12

**Authors:** Jihye Heo, Jin Lee, Hwamin Woo, Jihee Nam, Suna Kang, Hyunsoo Kim, Whanhee Lee, Kangmo Ahn, Danbee Kang, Eliseo Guallar, Sung Won Kang, Juhee Cho

**Affiliations:** 1 Department of Clinical Research Design and Evaluation, Samsung Advanced Institute for Health Sciences and Technology, Sungkyunkwan University, Seoul, South Korea; 2 Center for Clinical Epidemiology, Samsung Medical Center, Seoul, South Korea; 3 Korea Environment Institute, Sicheong-daero, Sejong, South Korea; 4 Research and Management Center for Health Risk of Particulate Matter, Seoul, South Korea; 5 School of Biomedical Convergence Engineering, Pusan National University, Yangsan, South Korea; 6 Environmental Health Center for Atopic Diseases, Samsung Medical Center, Seoul, South Korea; 7 Department of Pediatrics, Samsung Medical Center, Sungkyunkwan University School of Medicine, Seoul, South Korea; 8 Department of Epidemiology, New York University School of Global Public Health, New York, New York, United States of America; Kyung Hee University College of Medicine, KOREA, REPUBLIC OF

## Abstract

**Objectives:**

Although particulate matter (PM) exposure poses significant public health risks, previous research has focused on limited clinical areas. However, emerging evidence and pathological mechanisms of PM suggest that PM may exert broader systemic effects across a wide range of diseases. Therefore, we aim to identify and prioritize research questions to evaluate health impacts of PM exposure across various clinical specialties.

**Methods:**

A structured collaborative process was conducted between April and November 2024 in South Korea, incorporating systematic literature reviews, multidisciplinary expert discussions, and knowledge-sharing seminars. The primary outcomes were the identification of diseases potentially influenced by PM exposure and the development of corresponding research questions. The literature review synthesized more than 417 publications, including the U.S. Environmental Protection Agency’s integrated science assessment materials, a government-issued abstract compendium on PM covering 2010–2019, and studies published from 2020 to 2024 identified via a structured search. These were categorized by exposure duration (short- or long-term) and diseases outcome (incidence or progression). Prioritization was based on three criteria: pathological causality, clinical impact (public health burden), and feasibility using the Korea National Health Insurance Service (K-NHIS).

**Results:**

A total of 99 experts from epidemiology, data science, and 14 clinical specialties participated. The experts panel (mean age: 46.1 years; mean professional experience: 20.5 years) identified 211 research questions across 80 diseases. These were classified by disease outcome: disease incidence (short-term, 54; long-term, 64) and progression (short-term, 47; long-term, 46). Notably, several clinical areas such as ophthalmology, dermatology, and otolaryngology were underrepresented.

**Conclusion:**

This structured, multidisciplinary approach broadened the scope of PM-related clinical research beyond commonly studied clinical area. This scalable framework can be adapted in other regions with similar claims data systems to guide evidence-based research agendas and inform public health policies.

## Introduction

Air pollution, particularly particulate matter (PM) exposure, has long been recognized as a critical public health issue because of its pervasive impact on human health [1,2]. Both short- and long-term exposure to PM are linked to severe health outcomes, contributing to increased rates of mortality, hospitalization, and emergency room visits [1–4]. Consequently, much of the research on air pollution has focused on the general population, associating PM exposure with well-known “environmental diseases” such as cardiovascular, respiratory, autoimmune diseases/disorders, and cancer [5]. However, PM can infiltrate the systemic circulation, reach multiple organs, and trigger inflammatory responses that contribute to the development or progression of a wide range of diseases [6]. This suggests that the health impacts of PM may extend beyond the traditionally recognized conditions and environmental diseases. However, few studies have systematically examined a broad spectrum of health outcomes, while integrating multidisciplinary clinical expertise and accounting for the practical constraints of administrative claims data. Moreover, prior studies have primarily used a data-driven approach with population-based cohorts, often without considering the underlying pathological mechanisms or clinical feasibility [7]. As a result, our understanding of how PM exposure contributes to disease onset or influences the progression of pre-existing conditions across diverse clinical areas remains limited.

In Korea, there is the Korea National Health Insurance Service (K-NHIS) database, which contains comprehensive, population-wide health insurance claims data from South Korea’s single-payer healthcare system. Covering nearly the entire Korean population, the K-NHIS includes information on inpatient and outpatient visits, procedures, and prescriptions, enabling large-scale investigations into the effects of PM across diverse clinical conditions [8]. Identifying diseases that are particularly susceptible to the effects of PM exposure is a critical first step in mitigating its health burden. By clarifying which conditions are most influenced by PM and which populations are most vulnerable, targeted interventions can be implemented to reduce exposure and prevent disease onset or progression. However, given the broad scope of possible analyses, it is essential to prioritize research questions by specifying relevant exposure periods and outcome types to focus efforts on the most clinically meaningful and feasible investigations. This study aims to identify and prioritize research questions for evaluations of PM-related health effects by implementing a structured collaborative process with a multidisciplinary team.

## Methods

### Study design and participants (experts)

**Fig 1:**
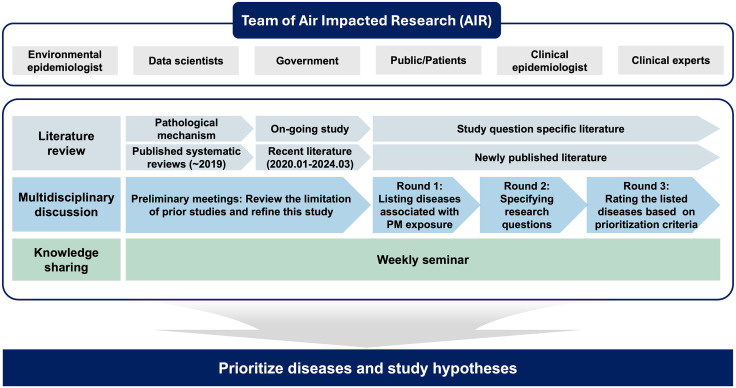
Structured multidisciplinary framework for prioritizing diseases and research questions related to PM exposure. This conceptual framework summarizes the AIR team’s collaborative process, including literature review, expert discussions, and stakeholder engagement, to generate and prioritize research questions for evaluating PM-related health effects.

This study employed a comprehensive approach with three components: a literature review, multidisciplinary discussion, and knowledge-sharing (**Fig 1**). The structured prioritization process was conducted between April and November 2024 as part of the AIR Impacted Research (AIR) study. Expert recruitment was conducted from April to June 2024. We invited clinical experts from departments associated with PM-related health outcomes, as well as leading researchers in environmental and clinical epidemiology in South Korea. To broaden the pool of experts, all invitees were asked to recommend colleagues with relevant expertise or research experience in PM exposure. We also invited presenters from major academic conferences, including the 2024 Sapporo Exposome Symposium and the 2024 Korean Society of Environmental Epidemiology. All invited experts provided informed consent to participate in this study. Consent was informed and obtained via email. Experts were given a detailed explanation of the study objectives, scope, and anticipated roles. Email responses confirming participation served as documentation of verbal consent. Additionally, we reviewed national disease statistics and insights from a national conference on PM and health to reflect the public and patient perspectives [9]. This study was approved by the Institutional Review Board of the Samsung Medical Center (No.2024-11-018).

### Prioritize diseases and research questions

To systematically prioritize diseases to evaluate the health impacts of PM exposure, we conducted a structured process consisting of a literature review, multidisciplinary discussion, and knowledge sharing.

#### Knowledge sharing.

Between May and September 2024, we held 23 weekly seminars to facilitate interdisciplinary knowledge exchange and identify gaps in PM-related research. The seminars were open to the AIR team and external researchers interested in the health impacts of PM.

#### Literature review.

A comprehensive literature review was conducted to examine the pathological mechanisms of PM and its associated health impacts. First, we reviewed a systematic report by the U.S. Environmental Protection Agency (EPA) that synthesized studies related to PM up to December 2019 [6]. We also reviewed the 2021 abstract collection on PM and air pollution jointly published by the Korea Disease Control and Prevention Agency and the Korean Academy of Medical Science, which summarizes the literature from 2010 to 2019 [10]. To ensure the inclusion of recent findings, we replicated the search strategy of the 2021 abstract collection to identify publications from January 2020 to March 2024 (S1 Table). Ongoing studies were identified using ClinicalTrials.gov (the search was conducted on April 17, 2024). The relevant literature on newly identified diseases was also reviewed through multidisciplinary discussions.

#### Multidisciplinary discussion.

We conducted three structured rounds of discussion to identify and prioritize the diseases. Preliminary meetings with environmental and clinical epidemiology experts were held to review the limitations of previous studies and refine the process.

##### Round 1: Listing diseases associated with PM exposure.

The AIR team included specialists in environmental epidemiology, data science, clinical epidemiology, various clinical departments, and government agencies. It included 14 clinical departments, oncology, respirology, cardiology, endocrinology, gastroenterology, immunology, nephrology, urology, gynecology, dermatology, otolaryngology, ophthalmology, psychology, and neurology, which participated in the disease prioritization process. Each group received a summary of the literature review and used both the summary and their clinical expertise to compile a list of diseases potentially influenced by PM exposure.

##### Round 2: Specifying research questions.

Experts were asked to specify research questions for each candidate disease, including the relevant exposure period (short- or long-term) and health outcomes (disease incidence or progression). Short-term exposure was defined as <1 month and long-term exposure as ≥1 month consistent with exposure-duration categories in the U.S. EPA’s human health risk assessment framework [11]. In this study, disease progression was defined as a deterioration in the clinical course of a pre-existing condition. This included worsening of symptoms or severity (progression), recurrence after remission (relapse), acute worsening of a chronic condition (exacerbation), and development of secondary conditions attributable to the pre-existing disease (complication). This definition encompasses any clinical event that may negatively affect patient prognosis or quality of life [12]. Responses were collected via email to encourage thoughtful and unprompted contributions tailored to disease-specific characteristics.

##### Round 3: Rating the listed diseases based on prioritization criteria.

Each expert independently evaluated the candidate diseases using a structured survey with predefined definitions for three criteria—causality, clinical impact, and feasibility—rating each criterion as high, medium, or low. Causality was defined as the strength and coherence of evidence linking PM exposure to the outcome, considering biological plausibility and the quality of epidemiologic/experimental study designs. Clinical impact referred to the public health burden of the disease, including its incidence, morbidity, and mortality, and expected impact on clinical decision making. Feasibility denoted the likelihood of identifying and analyzing the outcome using the Korean National Health Insurance Service (K-NHIS) database (e.g., International Classification of Diseases, 10th revision (ICD-10) coding accuracy, data completeness, and data availability). Ratings were submitted individually and collected electronically, without group discussion at this stage, to minimize bias. Individual assessments were subsequently aggregated for final prioritization.

Prioritization was determined based on expert ratings. For departments involving four or more experts, a disease was prioritized if 30% or more of the experts rated either causality or clinical impact as high. For departments with three or fewer experts, prioritization required at least two experts to rate either causality or clinical impact as high. All prioritized hypotheses were then evaluated for feasibility.

## Results

A total of 99 experts from 16 specialized areas participated in the study. The mean (standard deviation, SD) age was 46.1 (7.0) years, and 61.6% were male. Approximately 68.7% worked at tertiary hospitals, and the mean (SD) years of professional experience was 20.5 (7.4) years (Table 1). We hosted 23 seminars covering topics such as environmental and clinical epidemiology, air pollution research methods, PM mitigation strategies, animal models, and recent trends in PM-related studies (S2 Table).

**Table 1:** Characteristics of participating experts in the structured multidisciplinary prioritization process (N = 99).

Characteristics	N(%)
**Age, years**	46.1 (7.0)
**Sex, Male**	61 (61.6)
**Work experience, years**	20.5 (7.4)
**Organization, tertiary hospital**	68 (68.7)
**Specialty**	
Oncology	13 (13.1)
Respirology	13 (13.1)
Immunology	7 (7.1)
Neurology	7 (7.1)
Gynecology	6 (6.1)
Nephrology	6 (6.1)
Otolaryngology	6 (6.1)
Gastroenterology	6 (6.1)
Dermatology	6 (6.1)
Cardiology	4 (4.0)
Ophthalmology	4 (4.0)
Urology	4 (4.0)
Endocrinology	4 (4.0)
Psychology	3 (3.0)
Epidemiology & Data Science	10 (10.1)

Values are expressed as mean (SD) or N (%).

Research questions were categorized into four groups based on the PM exposure duration and outcome type: short-term exposure and disease incidence, short-term exposure and disease progression, long-term exposure and disease incidence, and long-term exposure and disease progression.

**Fig 2:**
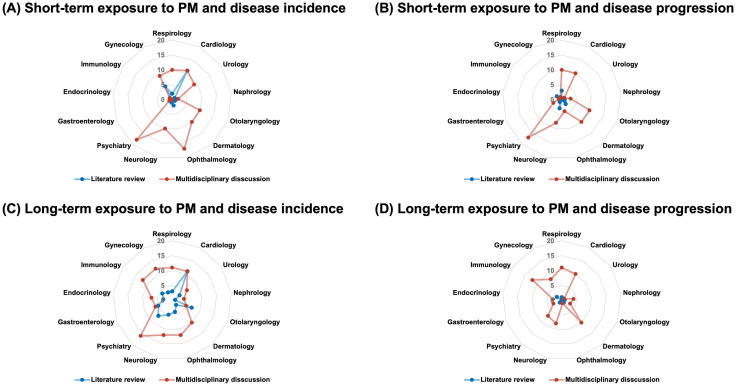
Comparison of diseases identified in the literature versus multidisciplinary expert discussions for PM-related health outcomes. This spider chart shows the number of diseases associated with short-term PM exposure and disease incidence, as identified in the literature (blue) and proposed during expert discussions (red). Notable increases in areas such as ophthalmology, dermatology, and psychiatry highlight underexplored domains in environmental health research.

As part of Round 1, we conducted a literature review to identify diseases previously studied in relation to PM exposure. This review revealed that only 24 and 58 diseases were investigated for disease incidence following short- and long-term PM exposure, respectively, while only 14 and 6 diseases were investigated for disease progression under the same exposure durations (Fig 2).

In Round 2, clinical experts were asked to specify research questions for each candidate disease, including the relevant exposure period and outcome type. As a result of this expert-driven process, the number of diseases considered increased substantially. Specifically, 108 and 124 diseases were identified as potentially associated with disease incidence following short- and long-term PM exposure, respectively. Similarly, 79 and 81 diseases were identified as potentially associated with disease progression for short- and long-term PM exposure, respectively. This expansion included disease areas that were underrepresented in the literature. For example, 18 psychiatric, 17 ophthalmologic, 10 dermatologic, and 10 otolaryngologic diseases were potentially associated with short-term exposure (**Fig 2, panel A**). Additionally, 12 gynecological and immunological diseases were associated with long-term exposure in the context of disease incidence (Fig 2**, panel C**). The oncology group concluded that cancer incidence was associated only with long-term PM exposure.

**Fig 3:**
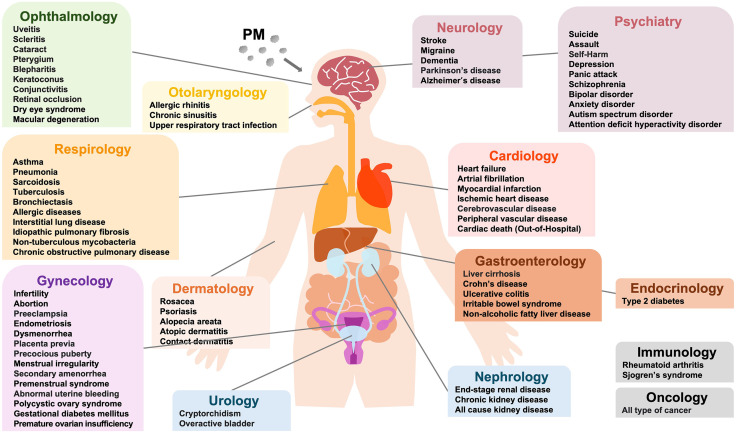
Prioritized diseases for PM-related health impact evaluation. This figure illustrates the diseases prioritized for further investigation based on structured expert discussion. Diseases are grouped by clinical specialty and reflect those identified as having potential associations with PM exposure, either in terms of disease incidence or progression.

Following Round 3 prioritization, 80 diseases were selected for further evaluation using the K-NHIS data (Fig 3). A total of 211 research questions were defined, including 54 related to disease incidence following short-term PM exposure, 47 related to disease progression following short-term PM exposure, 64 related to disease incidence following long-term PM exposure, and 46 related to long-term disease progression (Table 2). Among the 79 diseases that were not prioritized, the primary reasons for exclusion were limited feasibility based on K-NHIS data (35.4%), low causal evidence (32.9%), and low clinical impact (31.6%).

**Table 2:** Prioritized research questions on associations between PM exposure and clinical outcomes, with disease prioritization ratings.

Disease category	Selected	Short-term exposure		Long-term exposure		Rating for disease prioritization criteria		
		Incidence	Progression	Incidence	Progression	Causality	Clinical impact	Feasibility
**Respiratory**								
Idiopathic pulmonary fibrosis	O	O	O	O	O			
Tuberculosis	O	O	O	O	O			
Sarcoidosis	O	X	X	O	O			
Chronic obstructive pulmonary disease	O	O	O	O	O			
Asthma	O	O	O	O	O			
Pneumonia	O	O	O	O	O			
Bronchiectasis	O	O	O	O	O			
Interstitial lung diseases	O	O	O	O	O			
Non-tuberculous mycobacterium	O	O	O	O	O			
Allergic diseases	O	O	O	O	O			
Respiratory symptoms	X	O	O	O	O			
**Cardiology**								
Myocardial infarction	O	O	O	O	O			
Atrial fibrillation	O	O	O	O	O			
Out-of-Hospital cardiac arrest	O	O	O	O	O			
Cerebrovascular disease	O	O	O	O	O			
Heart failure	O	O	O	O	O			
Ischemic heart disease	O	O	O	O	O			
Peripheral vascular disease	O	O	X	O	O			
Arrhythmia	X	O	O	O	O			
Atherosclerosis	X	O	O	O	X			
Pulmonary thromboembolism	X	O	O	O	O			
Hypertension	X	O	O	O	O			
**Psychiatry**								
Suicide	O	O	O	X	O			
Autism spectrum disorder	O	O	O	O	X			
Schizophrenia	O	O	O	O	O			
Depression	O	O	O	O	O			
Panic attack	O	O	O	O	O			
Bipolar disorder	O	O	O	O	O			
Anxiety disorder	O	O	O	O	O			
Assault	O	O	O	O	X			
Attention deficit hyperactivity disorders	O	O	O	O	X			
Self-harm	O	O	O	O	X			
Substance use disorder	X	O	X	O	X			
Insomnia	X	O	O	O	O			
Tourette syndrome	X	O	O	O	X			
Tic disorder	X	O	O	O	X			
Eating disorder	X	O	O	O	X			
Obsessive compulsive disorder	X	O	O	O	X			
Post traumatic stress disorder	X	O	O	O	X			
Takotsubo syndrome	X	O	O	X	X			
**Nephrology**								
End-stage renal disease	O	X	O	O	O			
All cause kidney disease	O	O	O	O	O			
Chronic kidney disease	O	X	X	O	O			
Glomerulitis	X	X	X	O	O			
Acute kidney injury	X	O	O	X	X			
**Neurology**								
Alzheimer’s disease	O	X	O	O	O			
Dementia	O	O	O	O	O			
Stroke	O	O	O	O	O			
Parkinson’s disease	O	X	O	O	O			
Migraine	O	O	O	O	O			
Epilepsy	X	O	O	O	O			
Infectious diseases of the nervous system	X	O	X	O	X			
Neuroimmunological disease	X	O	X	O	X			
Syncope	X	O	X	O	X			
Delirium	X	O	X	O	X			
Chronic pain disorders	X	O	O	O	O			
Neuropsychiatric symptoms	X	O	O	O	O			
**Urology**								
Cryptorchidism	O	O	X	X	X			
Overactive bladder	O	O	X	O	X			
Benign prostatic hyperplasia	X	X	X	O	X			
Fetal hydronephrosis	X	O	X	X	X			
Hypospadias	X	O	X	X	X			
Congenital ureteropelvic junction obstruction	X	O	X	X	X			
Vesicoureteral reflux	X	O	X	X	X			
Interstitial cystitis	X	O	X	O	X			
Prostatitis	X	O	X	O	X			
Male infertility	X	X	X	O	X			
**Gynecology**								
Preeclampsia	O	O	X	X	X			
Gestational diabetes mellitus	O	O	X	X	X			
Placenta previa	O	O	X	X	X			
Abnormal uterine bleeding	O	X	X	O	X			
Endometriosis	O	X	X	O	X			
Infertility	O	X	X	O	X			
Polycystic ovary syndrome	O	X	X	O	X			
Abortion	O	O	X	X	X			
Precocious puberty	O	X	X	O	X			
Secondary amenorrhea	O	X	X	O	X			
Premature ovarian insufficiency	O	X	X	O	X			
Dysmenorrhea	O	X	X	O	O			
Premenstrual syndrome	O	X	X	X	O			
Menstrual irregularity	O	X	X	O	O			
Preterm premature rupture of membrane	X	O	X	X	X			
Stillbirth	X	O	X	X	X			
Pregnancy complications	X	O	O	X	X			
Ectopic pregnancy	X	O	X	X	X			
Birth defect	X	O	X	X	X			
Myoma	X	X	X	O	X			
Age at menarche	X	X	X	X	O			
Pre-menopause	X	X	X	X	O			
Age at menopause	X	X	X	O	O			
Menopausal symptoms	X	X	X	X	O			
Vasomotor symptoms	X	X	X	O	O			
**Dermatology**								
Psoriasis	O	O	O	O	O			
Rosacea	O	O	O	O	O			
Atopic dermatitis	O	O	O	O	O			
Contact dermatitis	O	O	O	O	O			
Alopecia areata	O	x	x	O	O			
Urticaria	X	O	O	O	O			
Seborrheic dermatitis	X	O	O	O	O			
Eczema	X	O	O	O	O			
Vitiligo	X	O	O	O	O			
**Immunology**								
Rheumatoid arthritis	O	X	X	O	O			
Sjogren’s syndrome	O	O	O	O	O			
Systemic lupus erythematosus	X	X	X	O	O			
Ankylosing spondylitis	X	X	X	O	O			
Comprehensive immune disease	X	X	X	O	O			
Systemic sclerosis	X	X	X	O	O			
Behcet’s disease	X	X	X	O	O			
Dermatomyositis	X	X	X	O	O			
Juvenile idiopathic arthritis	X	X	X	O	O			
Autoimmune hemolytic anemia	X	X	X	O	O			
Pernicious anemia	X	X	X	O	O			
Immune thrombocytopenic purpura	X	X	X	O	O			
**Endocrinology**								
Type 2 diabetes	O	X	O	O	O			
Hyperthyroidism	X	X	X	O	X			
Hypothyroidism	X	X	X	O	X			
Type 1 diabetes	X	X	X	O	X			
Grave’s disease	X	X	X	O	O			
Hashimoto’s thyroiditis	X	X	X	O	X			
**Otolaryngology**								
Upper respiratory tract infection	O	O	O	X	X			
Chronic sinusitis	O	O	O	O	X			
Allergic rhinitis	O	O	O	O	O			
Sinonasal inverted papilloma	X	X	X	O	O			
Anosmia	X	O	O	X	X			
Chronic tonsillitis	X	O	O	X	X			
Epistaxis	X	O	O	X	X			
Chronic rhinitis	X	O	O	X	X			
Chronic otitis media	X	X	X	O	O			
Facial paralysis	X	O	O	X	X			
Tinnitus	X	O	O	O	X			
Sudden sensorineural hearing loss	X	O	O	X	X			
**Gastroenterology**								
Crohn’s disease	O	X	O	O	O			
Ulcerative colitis	O	X	O	O	O			
Irritable bowel syndrome	O	X	X	O	X			
Non-alcoholic fatty liver disease	O	X	X	O	X			
Liver cirrhosis	O	X	O	X	X			
Eosinophilic esophagitis	X	O	X	O	X			
Celiac disease	X	X	X	O	O			
**Ophthalmology**								
Dry eye syndrome	O	O	X	X	X			
Conjunctivitis (allergic)	O	O	X	X	X			
Blepharitis	O	O	X	X	X			
Pterygium	O	X	X	O	X			
Keratoconus	O	X	X	O	X			
Cataract	O	X	X	X	O			
Retinal artery occlusion	O	O	X	O	X			
Retinal vein occlusion	O	O	X	O	X			
Non-infectious uveitis, anterior	O	O	O	X	X			
Non-infectious uveitis, non-anterior	O	O	O	X	X			
Non-infectious scleritis	O	O	O	X	X			
Age-related macular degeneration (overall)	O	O	X	O	X			
Age-related macular degeneration (exudative)	O	O	X	O	X			
Rhegmatogenous retinal detachment	X	O	X	O	X			
Vision-threatening diabetic retinopathy	X	O	X	O	X			
Optic neuritis	X	O	O	X	X			
Ischemic optic neuropathy	X	O	X	O	X			
Ocular motor cranial nerve palsy	X	O	X	O	X			
Glaucoma	X	O	X	O	X			
Keratitis	X	O	X	X	X			
Presbyopia	X	X	X	O	X			

Cell colors represent the proportion of “High” responses: (
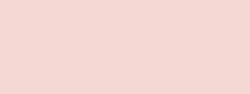
 (≤ 30%), 
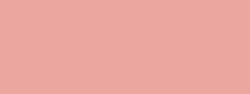
 (31%−60%), 
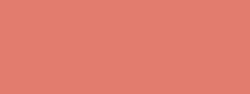
 (61%−90%), and 
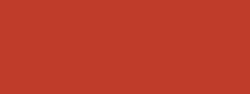
 (91%−100%).

## Discussion

This study applied a systematic and collaborative process to identify research priorities for assessing the health impacts of PM exposure using national health insurance claims data. By engaging almost 100 experts across diverse clinical specialties and applying structured prioritization criteria, we generated a broad set of research questions that extended our current understanding of PM-related health impacts beyond traditionally studied conditions.

A key contribution of this study lies in its methodological framework, which included a systematic literature review, iterative expert consultation, and claims data–oriented feasibility assessment. Unlike traditional environmental health research, which is often retrospective and focuses on a narrow set of disease areas, [2,13] our process was forward-looking, interdisciplinary, and designed to generate clinically relevant and data-feasible hypotheses. We bridged mechanistic plausibility with real-world analytical constraints by engaging 99 experts across 14 clinical specialties, alongside epidemiologists and data scientists. The use of structured rating criteria—pathological causality, clinical impact, and data feasibility—enabled a transparent prioritization process tailored to the K-NHIS claims data. The resulting 211 research questions and 80 prioritized diseases spanned a broad range of clinical domains, including those historically underrepresented in PM-related research. **Fig 2** illustrates the key gap between clinical priorities and existing literature on PM exposure. Whereas most published studies have focused on short-term impacts and disease incidence, clinical experts highlighted the need to investigate long-term exposure and disease progression, particularly in chronic and relapsing conditions. These areas are frequently rated as having high priority but low feasibility, reflecting challenges in longitudinal exposure assessments, residual confounding, and the limited ability of claims data to capture disease severity or progression. Addressing these gaps requires methodological innovation and data linkage, such as integrating electronic health records (EHRs), clinical registries, and advanced analytical approaches, to expand the scope of research and align it more closely with real-world clinical needs.

To contextualize these differences, the observed heterogeneity across the four exposure–outcome categories is expected and informative. Short-term incidence is more sensitive to acute PM-triggered events and care-seeking patterns, whereas long-term incidence/progression reflects cumulative exposure, latency, and disease-modifying pathways that are less readily captured in claims. In addition, measurement visibility differs by stratum: acute events are often observable through utilization spikes, while progression frequently requires clinical granularity (severity, function, biomarkers) that claims lack, leading to potential differential misclassification. By explicitly stratifying our prioritization, we (i) explain why scores legitimately differ across categories and (ii) derive stratum-specific guidance for future work (e.g., tailored exposure windows, refined outcome definitions, and registry/EHR linkages to enhance phenotyping).

Our study also underscores the importance of investigating diseases affecting organs directly exposed to PM, such as those in ophthalmology, dermatology, and otolaryngology. Direct contact with airborne particles may trigger localized oxidative stress, inflammation, and immune responses, [14] contributing to conditions such as dry eye syndrome, atopic dermatitis, and upper respiratory tract infections [15–17]. Although previous research primarily focused on systemic impacts, such as those involving the cardiovascular or immune systems, [5] our findings suggest that localized, symptomatic responses to PM exposure also warrant attention. Incorporating clinical insights into areas that are less commonly studied in environmental epidemiology may help identify overlooked health outcomes relevant to both clinical care and public health.

Lower prioritization scores for certain diseases require careful interpretation. These findings represent hypotheses rather than conclusions and reflect a structured evaluation process that considers data feasibility, pathological plausibility, and clinical impact. Certain conditions were deprioritized because of the inherent limitations of claims data, including underdiagnosis, subtle symptomatology, or low healthcare utilization, particularly in the context of certain mental health disorders [12,18]. In addition, claims data often lack detailed clinical information, including disease severity, functional status, and biomarker profiles [12]. These are crucial for assessing disease incidence and progression and establishing causal relationships with PM exposure. To address these gaps, future research should aim to integrate complementary data sources, including EHRs, clinical data warehouses, and national disease registries, to support more comprehensive and longitudinal analyses.

Our study presents a structured, criteria-based approach that combines clinical expertise, environmental epidemiology, and practical realities of real-world data. To our knowledge, few previous initiatives have applied such a comprehensive and methodologically transparent process to establish research priorities based on clinical impact and data feasibility. In particular, approximately 70% of the experts participating in our study were affiliated with tertiary hospitals, and their average clinical experience exceeded two decades, enhancing the credibility and depth of multidisciplinary discussions. This structured engagement model is adaptable to other countries with national claims systems or large health registries, such as those in the Nordic region, Taiwan, and parts of Europe, and may support the development of locally relevant research agendas and surveillance strategies. This integrated approach not only advances PM-related health research but also offers a transferable model for hypothesis generation in other health domains reliant on large-scale administrative data.

## Strengths and limitations

This study has several limitations. First, despite our efforts to recruit a diverse range of experts, certain clinical specialties were underrepresented, which may have influenced the breadth of the diseases considered. However, to mitigate this, we employed a snowball sampling strategy that allowed all invited participants to recommend additional experts without specialty restrictions. In addition, although our expert panel reflected multidisciplinary expertise to some extent, it did not include specialists whose primary focus is on artificial intelligence, machine learning, or multimodal data analysis. The inclusion of such experts may have enhanced the methodological diversity and depth of our findings. Future research will engage a broader range of experts from these rapidly advancing fields. Second, although expert-driven prioritization offers valuable clinical insight, the rating process may be influenced by individual judgement. To enhance consistency and transparency, we applied predefined and standardized definitions for pathological causality, clinical impact, and data feasibility, which were provided to all experts as guidance for assigning high/medium/low scores. Third, the use of a uniform 1-month threshold to distinguish short-term and long-term exposure across all disease outcomes. Although this choice was informed by standardized exposure-duration categories in environmental health research, including those defined by the U.S. EPA, disease latency and progression patterns vary substantially. Thus, a single threshold may oversimplify exposure-response relationship between PM exposure and health outcomes. Future studies should consider outcome-specific exposure windows and where feasible, evaluate alternative thresholds. Fourth, diseases were grouped by clinical systems based on the structured adopted in the U.S. EPA Integrated Science Assessment (ISA). While this provided a practical and standardized framework for organizing expert input and ensuring comparability with prior literature, it may not fully account for distinct biological mechanisms through which PM affects different diseases within the same category. Future work should consider mechanism-informed or phenotype-based groupings (e.g., inflammation-, autonomic-, or oxidative stress-mediated pathways) to better reflect within-system heterogeneity. Fifth, this study was conducted in the context of the South Korean healthcare system using national claims data; therefore, the findings may reflect country-specific clinical practices, environmental exposure patterns, and coding systems. Therefore, the generalizability to other settings should be approached with caution.

Nonetheless, this study was strengthened by its comprehensive and structured approach and by the engagement of nearly 100 experts with extensive clinical and research experience. By integrating expert input with a systematic literature review and emphasizing data feasibility, including consideration of claim codes and reimbursement practices, we developed a robust and replicable framework for prioritizing PM-related health research using administrative claims data. Importantly, the incorporation of multidisciplinary expert perspectives enabled the identification of clinically meaningful conditions that may not be fully captured in the existing literature. This approach allowed us to account for biological plausibility and real-world clinical relevance, complementing the evidence-based findings of the literature review. We also recognize a temporal gap between the literature synthesis and expert assessment: the review reflects evidence only up to the search end date and may lag due to publication/indexing delays, whereas expert input incorporates more current clinical observations. In addition, potential publication bias may limit the visibility of outcomes with null or non-significant findings, further underscoring the value of integrating evidence-based and expert-informed perspectives.

## Conclusion

This study presents a scalable, multidisciplinary framework for setting research agendas that align environmental health inquiries with the clinical relevance and practical constraints of large-scale administrative data systems. This approach is particularly valuable for countries with national claims databases or health registries, providing a transferable approach for supporting evidence generation to inform policy and public health actions. As the health impacts of environmental exposures become increasingly complex and context-specific, the adoption of transparent, interdisciplinary, and data-integrated strategies is essential for advancing clinically meaningful and data-feasible environmental health research.

## Supporting information

S1 Table Search term to ensure up-to-date literature. (DOCX)

S2 Table List of seminar topics. (DOCX)
