# Supplementary material for: Framework to prioritize health outcomes of particulate matter exposure using national claims data
Source: PLoS One. 2025 Dec 12;20(12):e0336511. doi: 10.1371/journal.pone.0336511 (PMC12700451; doi:10.1371/journal.pone.0336511)
Supplement: S1 Table — (DOCX) [file pone.0336511.s001.docx]

**Supplementary Appendix**

**Title: Framework to Prioritize Health Outcomes of Particulate Matter Exposure Using National Claims Data**

**S1 Table. Search term to ensure up-to-date literature**

| **Search** | **Query** | **Results** |
| --- | --- | --- |
| #1 | "Air Pollution"[Mesh] | 72,658 |
| #2 | "Air Pollution"[TI] OR "Air Pollutions"[TI] OR "air contamination"[TI] OR "atmosphere pollution"[TI] OR "atmospheric pollution"[TI] OR "polluted air "[TI] OR "polluted atmosphere"[TI] OR "pollution, air"[TI] OR "air pollutant "[TI] OR "air pollutants"[TI] | 20,116 |
| #3 | "Particulate Matter"[MeSH] | 82,308 |
| #4 | Particulate Matter*[TI] | 8,435 |
| #5 | #1 OR #2 OR #3 OR #4 | 128,738 |
| #6 | #5 NOT (Case Reports[ptyp] OR Comment[sb] OR Editorial[ptyp] OR Letter[ptyp] OR Case Report*[TI] Case series*[TI] OR Comment*[TI] OR Letter*[TI]) | 128,198 |
| #7 | #6 AND ("2020/01/01"[PDAT] : "2024/03/31"[PDAT]) AND English[lang] | 27,619 |
| #8 | #7 AND (Systematic[sb] OR "meta-analysis"[ptyp] OR systematic[TI] OR literature review*[TI] OR meta-analys*[TI] OR Randomized Controlled Trial[ptyp] OR Random*[TI] OR "cohort studies"[MeSH] OR Cohort[TIAB] OR "cross-sectional studies"[MeSH] OR Cross-section*[TI]) | 4,826 |
| #9 | #8 NOT ("animal"[MeSH] AND "mice"[MeSH]) | 415 |
