# Supplementary material for: Framework to prioritize health outcomes of particulate matter exposure using national claims data
Source: PLoS One. 2025 Dec 12;20(12):e0336511. doi: 10.1371/journal.pone.0336511 (PMC12700451; doi:10.1371/journal.pone.0336511)
Supplement: S2 Table — (DOCX) [file pone.0336511.s002.docx]

**Supplementary Appendix**

**Title: Framework to Prioritize Health Outcomes of Particulate Matter Exposure Using National Claims Data**

**S2 Table. List of seminar topics**

| **Topic of seminar** | **Date** |
| --- | --- |
| Health impact assessment study design of particulate matter: Study methodology of short-term impact assessment | 17 May 2024 |
| Cohort study on particulate matter and disease occurrence using the National Health Insurance Service data | 22 May 2024 |
| Particulate matter and Health impact | 31 May 2024 |
| Trends in research on particulate matter related to respiratory diseases | 7 Jun 2024 |
| Particulate matter and atopic dermatitis | 14 Jun 2024 |
| Status and challenges of particulate matter research | 21 Jun 2024 |
| Environmental-Pollution-Induced Neurological Effects (EPINEF) cohort study | 28 Jun 2024 |
| Methodology for long-term effects of particulate matter | 3 Jul 2024 |
| Latest trends in particulate matter health impact assessment | 5 Jul 2024 |
| Particulate matter and cardiovascular disease | 10 Jul 2024 |
| Environmental research using the National Health Insurance Service data | 12 Jul 2024 |
| Area Deprivation Index (ADI) in environmental research | 17 Jul 2024 |
| Particulate matter and mortality of chronic obstructive pulmonary diseases | 19 Jul 2024 |
| Assessing indoor particulate matter exposure for health impact assessment | 26 Jul 2024 |
| Particulate matter and pediatric cancer | 2 Aug 2024 |
| Particulate matter and urologic diseases: Infertility, bladder and kidney disease in animal models | 9 Aug 2024 |
| The Role of Human Aging in Linking Environmental Exposures and Brain Health | 16 Aug 2024 |
| The Science of Avoiding Particulate Matter Through Behavioral Interventions | 23Aug 2024 |
| Prenatal Exposure to Endocrine Disrupting Chemicals in Association with Autism Spectrum Disorder | 30 Aug 2024 |
| Health Impacts of Climate Change and Air Pollution on the Disable | 6 Sep 2024 |
| Exposure and Risk Assessment of Environmental Pollutants in the Era of Big Data and AI | 13 Sep 2024 |
| Understanding the Impact of Particulate Matter and Eye Disease | 20 Sep 2024 |
| Particulate Matter and Gastrointestinal Disease | 27 Sep 2024 |
